# Bilateral Ptosis, Diplopia, and Vertical Gaze Palsy: An Unusual Presentation of Adolescent Aquaporin-4 (AQP4)-Antibody Positive Neuromyelitis Optica Spectrum Disorder

**DOI:** 10.7759/cureus.92051

**Published:** 2025-09-11

**Authors:** Raya Flayyih, Abdalqader Abujouda, Waseem Fathalla

**Affiliations:** 1 Pediatric Residency Program, Department of Pediatrics, Sheikh Shakhbout Medical City Abu Dhabi, Abu Dhabi, ARE; 2 Pediatric Residency Program, Department of Pediatrics, Education Institute, Shiekh Khalifa Medical City Hospital, Abu Dhabi, ARE; 3 Pediatric Neurology, Sheikh Shakhbout Medical City Abu Dhabi, Abu Dhabi, ARE

**Keywords:** aquaporin-4 antibody, bilateral ptosis, neuromyelitis optica igg, neuromyelitis optica-igg, neuromyelitis optica spectrum disorder, transverse myelitis

## Abstract

Atypical presentations of neuromyelitis optica spectrum disorder (NMOSD) continue to expand in pediatrics. NMOSD is mediated by pathogenic antibodies against aquaporin-4 (AQP4-IgG), a water channel protein found on astrocytes. NMOSD is distinct from multiple sclerosis in both its clinical course and treatment response. Our objective is early recognition for prevention of long-term neurological damage.

We report a 14-year-old previously healthy girl who presented with subacute bilateral ptosis, diplopia, and vertical gaze palsy. Brain MRI revealed bilateral, nearly symmetric areas of T2 hyperintensity in the posteromedial thalami and periaqueductal region of the midbrain. Cerebrospinal fluid analysis was unremarkable; however, aquaporin-4 (AQP4) antibodies were detected in the cerebrospinal fluid (CSF) using a fluorescence-activated cell sorting (FACS) based assay at a titer of 1:128. She was treated with intravenous methylprednisolone and thiamine, followed by an oral steroid taper, with marked clinical improvement. This case highlights an atypical, brainstem-predominant presentation of NMOSD in a pediatric patient. It emphasizes the variability of NMOSD presentations in children and underscores the importance of early, comprehensive workup in patients with evolving neuro-ophthalmic symptoms. The purpose of this report is to raise clinical awareness of uncommon NMOSD presentations that may mimic other conditions and delay diagnosis.

## Introduction

Neuromyelitis optica spectrum disorder (NMOSD) is a rare, relapsing autoimmune demyelinating condition of the central nervous system that primarily targets the optic nerves, spinal cord, and sometimes the brainstem and hypothalamus [1]. It is mediated by pathogenic immunoglobulin G antibodies against aquaporin-4 (AQP4-IgG), a water channel protein densely expressed on astrocytes [2]. Clinically, NMOSD typically presents with acute episodes of optic neuritis, longitudinally extensive transverse myelitis (LETM), area postrema syndrome (persistent nausea, vomiting, or hiccups), or hypothalamic involvement, especially in pediatric cases [3]. Diagnosis is confirmed by detecting AQP4-IgG antibodies; seropositivity is ~99% sensitive and 90% specific [4], via a cell-based assay in the presence of at least one core clinical feature and MRI findings that exclude alternative causes such as multiple sclerosis, according to the 2015 international consensus criteria [3].

MRI usually reveals lesions involving ≥3 vertebral segments in the spinal cord or optic chiasm involvement [5]. Cerebrospinal fluid (CSF) analysis may show pleocytosis but typically lacks oligoclonal bands [6]. Early initiation of immunosuppressive therapy is critical, with acute attacks managed using high-dose intravenous corticosteroids or plasma exchange, followed by long-term immunosuppression (e.g., rituximab, azathioprine, or mycophenolate) to reduce relapse risk and progression [7]. Prompt recognition and treatment are essential to prevent irreversible neurological disability, especially in pediatric patients with atypical presentation [3].

Early differentiation of NMOSD from multiple sclerosis (MS) is of paramount importance, as NMOSD, especially the relapsing variant, has more severe morbidity than MS [8]. In addition, the approach to treatment (immunosuppression, anti-B-cell therapy) is different from MS, and some disease-modifying therapies used for MS may even be harmful in NMOSD [9]. 

## Case presentation

A 14-year-old previously healthy female presented with one month of progressive ocular symptoms beginning with bilateral eye redness and swelling, initially presumed to be allergic conjunctivitis following exposure to sandy weather. She was prescribed nonspecific anti-allergic eye drops with no improvement. Symptoms progressed over one week to persistent periocular swelling, diplopia, and restricted vertical eye movements, accompanied by bilateral ptosis. She reported no headache, painful eye movements, nausea, vomiting, dysphagia, respiratory difficulty, motor or sensory changes, or sphincter disturbance. There was no reported visual acuity loss, although she had recently been prescribed corrective eyeglasses. She experienced decreased appetite over the preceding three months but no other systemic symptoms. There is a strong family history of autoimmune diseases such as Crohn's disease, systemic lupus erythematosus, and hypothyroidism affecting her brother, sister, and one of her cousins, respectively.

**Figure 1:**
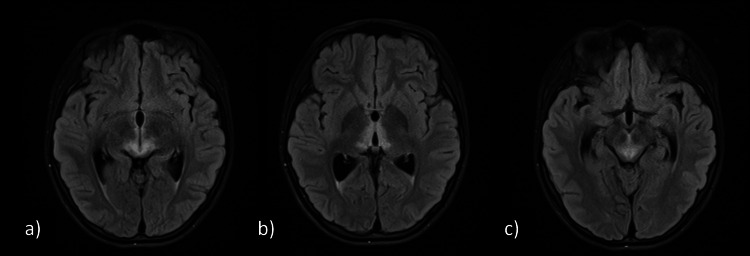
Flair axial MRI brain MRI: Magnetic resonance imaging Bilateral nearly symmetric areas of signal intensity in the posterior (a) and medial (b) thalami as well as the periaqueductal region of the midbrain (c). No significant interval change compared to the previous scan done on 4 May 2025. No abnormal signal identified in the basal ganglia, cerebral or cerebellar white matter, or optic pathways. The orbits appear unremarkable. No signs of optic nerve signal abnormality or atrophy. No hydrocephalus or midline shift. No abnormal intracranial enhancement.

Brain MRI confirmed hyperintense T2 signals involving the medial thalamus and periaqueductal region of the midbrain (Figure 1), raising concern for metabolic encephalopathy. She was empirically treated with thiamine with subjective improvement. However, given the lack of encephalopathy or other systemic involvement, she presented it to our emergency department for further evaluation.

On examination, she was alert, with no distress. Cranial nerve examination revealed anisocoria (left pupil 4 mm, right 3 mm), bilateral ptosis, and near-complete vertical gaze palsy with upward gaze nystagmus. Horizontal gaze was preserved but associated with horizontal diplopia. Fundoscopic examination showed sharp optic discs bilaterally. The sensation and motor function of the face were intact. Hearing, speech, and palate elevation were normal. Tongue and shoulder girdle strength were preserved. The motor exam showed normal tone, strength (5/5), and reflexes (+1). Gait and cerebellar testing were normal. No abnormal movements were noted. The remainder of the physical examination, including assessment for neurocutaneous stigmata, was unremarkable.

A comprehensive neurological workup was performed. CSF analysis showed normal cell count, glucose, and protein; cultures and the BioFire (bioMérieux, Marcy-l'Étoile, France) panel were negative. The autoimmune encephalitis panel was unremarkable.

CSF NMO/AQP4 antibody was positive by FACS-based assay at a titer of 1:128 (reference <1:2), supporting the diagnosis of neuromyelitis optica spectrum disorder.

Serum myelin oligodendrocyte glycoprotein (MOG) antibody, anti-MuSK, anti-AChR antibodies, and CSF IgG index were within normal limits. Patterned visual evoked potentials (VEP) showed delayed P100 latencies bilaterally (120 and 119 ms), consistent with demyelination of the visual pathways. Nerve conduction studies were normal.

**Figure 2:**
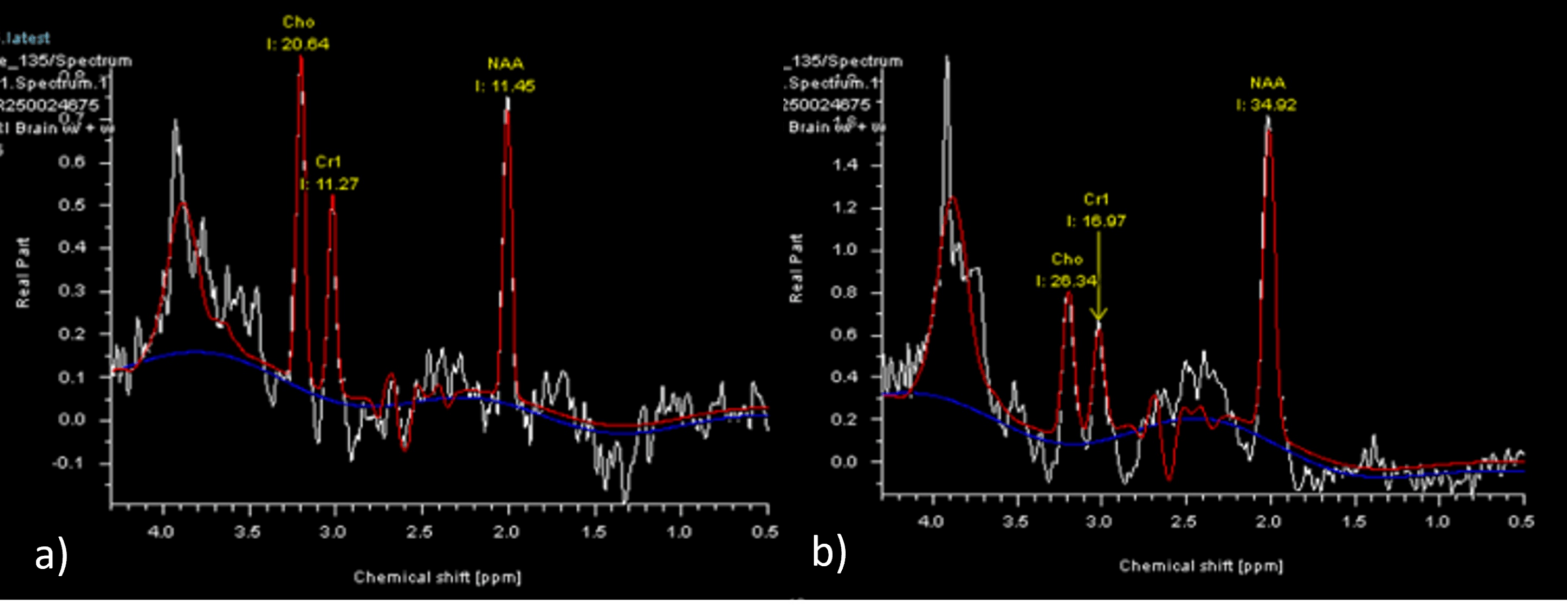
MR spectroscopy affected area of midbrain/thalamus voxel (left figure) Patient’s MR spectroscopy (a) demonstrating elevated choline (Cho) peak, with mildly reduced N-acetylaspartate (NAA) and preserved creatine (Cr), inverted doublet at \R\1.3 ppm, consistent with the presence of lactate. Control MR spectroscopy of a normal patient for comparison (b).

MR spectroscopy of the affected thalamic/tectal region demonstrated elevated choline, mildly reduced N-acetylaspartate, preserved creatine, and a lactate peak at 1.3 ppm, suggestive of chronic gliosis or demyelination (Figure 2).

The metabolic profile suggests chronic gliosis or demyelination in the sampled region.

The patient was treated with a five-day course of intravenous methylprednisolone 30 mg/Kg/day alongside high-dose intravenous thiamine 200 mg every 8 hours. Notable improvement in bilateral ptosis was observed, and she was subsequently discharged on oral thiamine and a tapering regimen of oral prednisolone (starting at 35 mg daily). On follow-up in clinic, she demonstrated significant clinical improvement, including complete resolution of ptosis and partial improvement in vertical gaze palsy. On her three-week follow-up visit, diplopia had diminished, and her neurological examination was otherwise normal. She developed mild steroid-induced acne during treatment, which was managed conservatively.

## Discussion

We highlight the atypical presentation of pediatric NMOSD, which is uncommon and often presents with atypical features that can resemble more benign conditions, leading to diagnostic delays [3]. This patient presented to us with a relapsing demyelinating illness, but the presenting features were neither optic neuritis nor myelitis.

The patient’s early symptoms, including eye redness and swelling, were misleading and led to initial empirical treatment with antihistamines and later IV thiamine, without improvement. Progression to bilateral ptosis, diplopia, and vertical gaze palsy suggested cranial nerve or brainstem involvement [5]. 

Brain MRI revealed hyperintense T2 signals involving the medial thalami and periaqueductal region of the midbrain. This radiological pattern raised concern for several possible etiologies. The differential diagnosis included metabolic or mitochondrial cytopathies such as thiamine deficiency, pyruvate dehydrogenase complex disorders, POLG-related disease, and Kearns-Sayre syndrome; autoimmune or demyelinating conditions, including neuromyelitis optica spectrum disorder (NMOSD), MOG-antibody-associated disease, and multiple sclerosis; and, less commonly, inflammatory conditions such as Bickerstaff brainstem encephalitis or collagen vascular disorders.

In our case, cerebrospinal fluid analysis was unremarkable, but aquaporin-4 (AQP4) antibodies were detected in the CSF using a FACS-based assay at a titer of 1:128. This seropositivity confirmed the diagnosis of NMOSD according to the 2015 international consensus criteria [3]. 

According to the revised diagnostic criteria by the international panel for NMO diagnosis criteria in 2015, lesions located in the area postrema, diencephalon, brainstem, and cerebrum, in addition to the spinal cord and optic nerve, are considered [3]. The detection of the AQP-4 antibody and a core clinical characteristic involving any of the above six regions, with the exclusion of other diseases, is sufficient to make the diagnosis of NMOSD. Diagnosis can be made even without antibody positivity if two of the above six sites are involved, with one of them at least being optic neuritis, LETM, or area postrema syndrome [10] (Tables 1, 2).

**Table 1:** International panel for NMOSD diagnostic criteria (2015) for NMOSD with AQP4-IgG NMOSD: neuromyelitis optica spectrum disorder, AQP4-IgG: pathogenic antibodies against aquaporin-4

Requirements	Core Clinical Characteristics
At least one core clinical characteristic	Optic neuritis
Positive serum test for AQP4-IgG	Acute myelitis
No better explanation (consider clinical and MRI red flags)	Area postrema syndrome (nausea, vomiting, hiccups)
	Other brainstem syndromes
	Symptomatic narcolepsy or acute diencephalic syndrome with MRI lesion(s)
	Symptomatic cerebral syndrome with MRI lesion(s)

**Table 2:** International panel for NMOSD diagnostic criteria (2015) for NMOSD without AQP4-IgG (or unavailable) NMOSD: neuromyelitis optica spectrum disorder, AQP4-IgG: pathogenic antibodies against aquaporin-4

Requirements	Details
At least two core clinical characteristics, all of the following:	• One must be optic neuritis, myelitis, or area postrema syndrome • Dissemination in space (e.g., ON + TM, ON + AP syndrome) • Note: isolated recurrent ON or recurrent TM do not qualify
Additional MRI requirements	• AP syndrome: dorsal medulla lesion • Myelitis: LETM (longitudinally extensive transverse myelitis) • ON: normal brain MRI OR ≥1/2 optic chiasm lesion
AQP4-IgG status	Negative test using best available assay, or testing unavailable
Exclusion	No better explanation for the clinical syndrome

Lumbar puncture findings, clear fluid, normal protein, normal glucose, and absence of oligoclonal bands further supported NMOSD and helped differentiate it from multiple sclerosis, which typically shows positive oligoclonal bands [6]. The patient’s significant response to high-dose intravenous steroids followed by oral taper is consistent with current first-line management for acute NMOSD attacks [7].

An important consideration in this case is the strong family history of autoimmune disease, which may reflect a genetic predisposition to immune dysregulation [11]. While NMOSD itself is usually sporadic, its coexistence with conditions such as Crohn’s disease and suspected lupus in close relatives raises the question of shared immunogenetic pathways [12].

This case underscores the importance of early recognition of atypical NMOSD presentations in children. Clinicians should maintain a high index of suspicion when common ophthalmologic symptoms fail to respond to conventional treatment, particularly in patients with evolving neurological signs or a family history of autoimmune disease.

## Conclusions

This case highlights the diagnostic challenges of neuromyelitis optica spectrum disorder (NMOSD) in pediatric patients, especially when initial symptoms mimic benign conditions such as allergic conjunctivitis. The progression from nonspecific ocular symptoms to brainstem involvement underscores the importance of maintaining a high index of suspicion in children with evolving neuro-ophthalmic signs. Early MRI imaging and prompt testing for AQP4-IgG antibodies are critical for diagnosis. Furthermore, this case emphasizes the importance of considering NMOSD in the differential diagnosis of atypical cranial nerve presentations, particularly in individuals with a family history of autoimmune diseases. Timely initiation of immunosuppressive therapy significantly improved the patient’s outcome.
